# Sepsis-Associated Coagulopathy Predicts Hospital Mortality in Critically Ill Patients With Postoperative Sepsis

**DOI:** 10.3389/fmed.2022.783234

**Published:** 2022-02-15

**Authors:** Chao Ren, Yu-xuan Li, De-meng Xia, Peng-yue Zhao, Sheng-yu Zhu, Li-yu Zheng, Li-ping Liang, Ren-qi Yao, Xiao-hui Du

**Affiliations:** ^1^Translational Medicine Research Center, Fourth Medical Center and Medical Innovation Research Division of the Chinese PLA General Hospital, Beijing, China; ^2^Department of Pulmonary and Critical Care Medicine, Beijing Chaoyang Hospital, Capital Medical University, Beijing, China; ^3^Department of General Surgery, First Medical Center of the Chinese PLA General Hospital, Beijing, China; ^4^Department of Emergency, Changhai Hospital, Naval Medical University, Shanghai, China; ^5^Department of Orthopedics, The Naval Hospital of Eastern Theater Command of People's Liberation Army of China, Zhoushan, China; ^6^Guangmingqiao Clinic, East Beijing Medical Area of the Chinese PLA General Hospital, Beijing, China; ^7^Department of Burn Surgery, Changhai Hospital, Naval Medical University, Shanghai, China

**Keywords:** sepsis, coagulopathy, intensive care unit, postoperative, mortality

## Abstract

**Background:**

The incidence of coagulopathy, which was responsible for poor outcomes, was commonly seen among patients with sepsis. In the current study, we aim to determine whether the presence of sepsis-associated coagulopathy (SAC) predicts the clinical outcomes among critically ill patients with postoperative sepsis.

**Methods:**

We conducted a single-center retrospective cohort study by including patients with sepsis admitted to surgical ICU of Chinese PLA General Hospital from January 1, 2014 to December 31, 2018. Baseline characteristics and clinical outcomes were compared with respect to the presence of SAC. Kaplan-Meier analysis was applied to calculate survival rate, and Log-rank test was carried out to compare the differences between two groups. Furthermore, multivariable Cox and logistic and linear regression analysis were performed to assess the relationship between SAC and clinical outcomes, including hospital mortality, development of septic shock, and length of hospital stay (LOS), respectively. Additionally, both sensitivity and subgroup analyses were performed to further testify the robustness of our findings.

**Results:**

A total of 175 patients were included in the current study. Among all included patients, 41.1% (72/175) ICU patients were identified as having SAC. In-hospital mortality rates were significantly higher in the SAC group when compared to that of the No SAC group (37.5% vs. 11.7%; *p* < 0.001). By performing univariable and multivariable regression analyses, presence of SAC was demonstrated to significantly correlate with an increased in-hospital mortality for patients with sepsis in surgical ICU [Hazard ratio (HR), 3.75; 95% Confidence interval (CI), 1.90–7.40; *p* < 0.001]. Meanwhile, a complication of SAC was found to be the independent predictor of the development of septic shock [Odds ratio (OR), 4.11; 95% CI, 1.81–9.32; *p* = 0.001], whereas it was not significantly associated with prolonged hospital LOS (OR, 0.97; 95% CI, 0.83–1.14; *p* = 0.743).

**Conclusion:**

The presence of SAC was significantly associated with increased risk of in-hospital death and septic shock among postoperative patients with sepsis admitted to ICU. Moreover, there was no statistical difference of hospital LOS between the SAC and no SAC groups.

## Introduction

Sepsis is a complex disorder caused by a dysregulated host response to infection. It poses great threats on the survival of patients admitted to intensive care units (ICUs) (1). Both morbidity and mortality of sepsis remain high, making it one of the leading causes of death in ICU globally (1, 2). Early recognition and prompt interference are mainstays to improve clinical outcomes for septic patients. Large-scale epidemiological studies documented that surgical patients accounted for approximately 30% of all sepsis patients in the United States, and the number of patients who developed postoperative sepsis were reported to elevate annually (3–5).

The incidence of coagulopathy, which was reportedly responsible for poor outcomes, was commonly seen among septic patients (6, 7). Sepsis-associated coagulopathy (SAC) is characterized by a prolonged international normalized ratio (INR) and reduced platelet counts, which can be attributed to the elevated level of tissue factors on the surface of the circulating endothelial cells and the impaired counterbalance between anticoagulant and fibrinolytic pathways under septic exposure (8). These alterations of the coagulation pathway cause an increased coagulant activity and a decreased fibrinolysis during sepsis progression, thereby resulting in fibrin deposition in the microcirculation and subsequent tissue ischemia (9).

Since surgery inevitably brings about bleeding and derangement to the hemostatic system, it is a major cause for the development of coagulopathy (10, 11). Meanwhile, increasing evidence demonstrate that coagulopathy in the postoperative period could result in excessive bleeding, higher transfusion requirements, and increased mortality rate (12, 13). Nevertheless, the development of sepsis also augments the effect of surgical insults on hemostatic system, which might be responsible for intractable coagulopathy. A groundbreaking study by Lyons et al. (7) revealed that the development of SAC was significantly associated with a higher hospital mortality rate, with patients possessing more severe SAC, thereby having a higher risk of in-hospital death. However, there were few studies that specifically addressed the relationship between SAC and clinical outcomes among postoperative patients with sepsis. Therefore, we carried out a retrospective study that specifically includes critically ill patients with postoperative sepsis to determine whether the presence of SAC predicted clinical outcomes, including in-hospital mortality, septic shock, and length of hospital stay (LOS).

## Methods

### Study Design and Population Selection

This study was a retrospective, single-center study conducted in the surgical ICU of Chinese PLA General Hospital, a tertiary care hospital located in Beijing. We included adult patients (aged > 18 years) with a diagnosis of sepsis upon ICU admission from January 1, 2014 to December 31, 2018. Patients were who (1) stayed in the SICU <48 h; (2) had preexisting coagulopathy; (3) received blood transfusion within the last 3 months; (4) had intake of anticoagulants, antiplatelet agents, antithrombotics, and thrombolytics within 1 week prior to ICU admission, with the exception of deep venous thrombosis (DVT) prophylaxis and a daily dosage of aspirin for cardiac prophylaxis; (5) had end-stage of liver diseases; and/or (6) had other diseases, including idiopathic thrombocytopenic purpura (ITP), multiple myeloma, hemolytic uremic syndrome, which could affect function and counts of platelets, were excluded.

### Data Collection

Clinical data on ICU admission of all participants were collected from the electronic patient record (EPR) system using a predesigned data collection form which included age, gender, body mass index (BMI), and comorbidities, including hypertension, diabetes, coronary heart disease (CHD), cerebral infarction, chronic obstructive pulmonary disease (COPD), chronic renal insufficiency (CRI), chronic heart failure (CHF), and malignant neoplasm. In addition, laboratory findings and results of blood gas analysis upon ICU admission were recorded from the EPR system. The use of clinical interventions during ICU stay was also obtained, including mechanical ventilation, renal replacement therapy, tracheotomy, deep vein catheterization, and blood transfusion. Furthermore, we also obtained a sequential organ failure assessment (SOFA) and Acute Physiology and Chronic Health Evaluation (APACHE) II scores upon ICU admission for evaluating the severity of illness.

### Definition

Sepsis-associated coagulopathy (SAC) was defined as (1) having an INR ≥1.4 without other known etiology (e.g., ITP, multiple myeloma) and (2) a platelet count of ≤150 × 10^9^/L or that with a decrease >30% within 24 h (14). Of note, patients with platelet counts ≤30 × 10^9^/L were not deemed as having SAC. Meanwhile, we stratified all patients with SAC into three levels of severity based on INR value. Particularly, mild SAC was defined as having an INR ≥1.4 and <1.6, moderate SAC was characterized by an INR ≥1.6 but <1.8, and, lastly, patients with INR ≥1.8 were deemed as having severe SAC.

Both sepsis and septic shock were diagnosed in line with the Third International Consensus Definitions for Sepsis and Septic Shock (Sepsis 3.0) criteria, in which sepsis was defined with a SOFA score ≥ 2 along with a confirmed or suspected infection. Patients complicated with septic shock was identified by the requirement of vasopressors in maintaining mean arterial pressure (MAP) of 65 mmHg and serum lactate level >2 mmol/L despite adequate fluid resuscitation.

### Outcome Measurements

The primary outcome of the current study was the in-hospital mortality, which was defined as the survival status upon hospital discharge. Secondary outcomes included the development of septic shock and hospital LOS.

### Statistical Analysis

Baseline characteristics were presented and compared between patients with and without SAC, using either Student *t*-test, Mann-Whitney *U* test, or Chi-square test, as appropriate. Categorical or ranked data were reported as count and proportion, while continuous variables were presented as median [interquartile range (IQR)] or mean [standardized differences (SD)].

Kaplan-Meier analysis was applied to calculate the survival rate, and Log-rank test was carried out to validate the statistical differences between the two groups. Cox regression model was adopted to investigate the association between the presence of SAC and the in-hospital mortality, whereas logistic regression model was applied to estimate whether SAC was associated with the incidence of septic shock. Linear regression analysis was used to assess the correlation between SAC and hospital LOS, for which odds ratios (ORs) were presented using the formula *OR* = *e*^β^*i*. Univariable analyses were initially performed, in which potentially confounding variables were selected based on *p* < 0.1. Thereafter, multivariable regression analysis was subsequently performed to confirm the independent association of SAC with clinical outcomes adjusting for confounding variables. A two-sided *P*-value of <0.05 was regarded as statistically significant. To further validate the robustness of our findings, we performed a series of subgroup and sensitivity analyses on our primary endpoints, stratifying cohort by INR value, age, gender, BMI, and platelet count.

All statistical analyses were performed using SPSS software (version 26; IBM Corporation, St. Louis, Missouri, USA) and R software (version.3.6.1; The R Project for Statistical Computing, TX, USA; http://www.r-project.org).

## Results

### Selection and Characteristics of Patients

**Figure 1:**
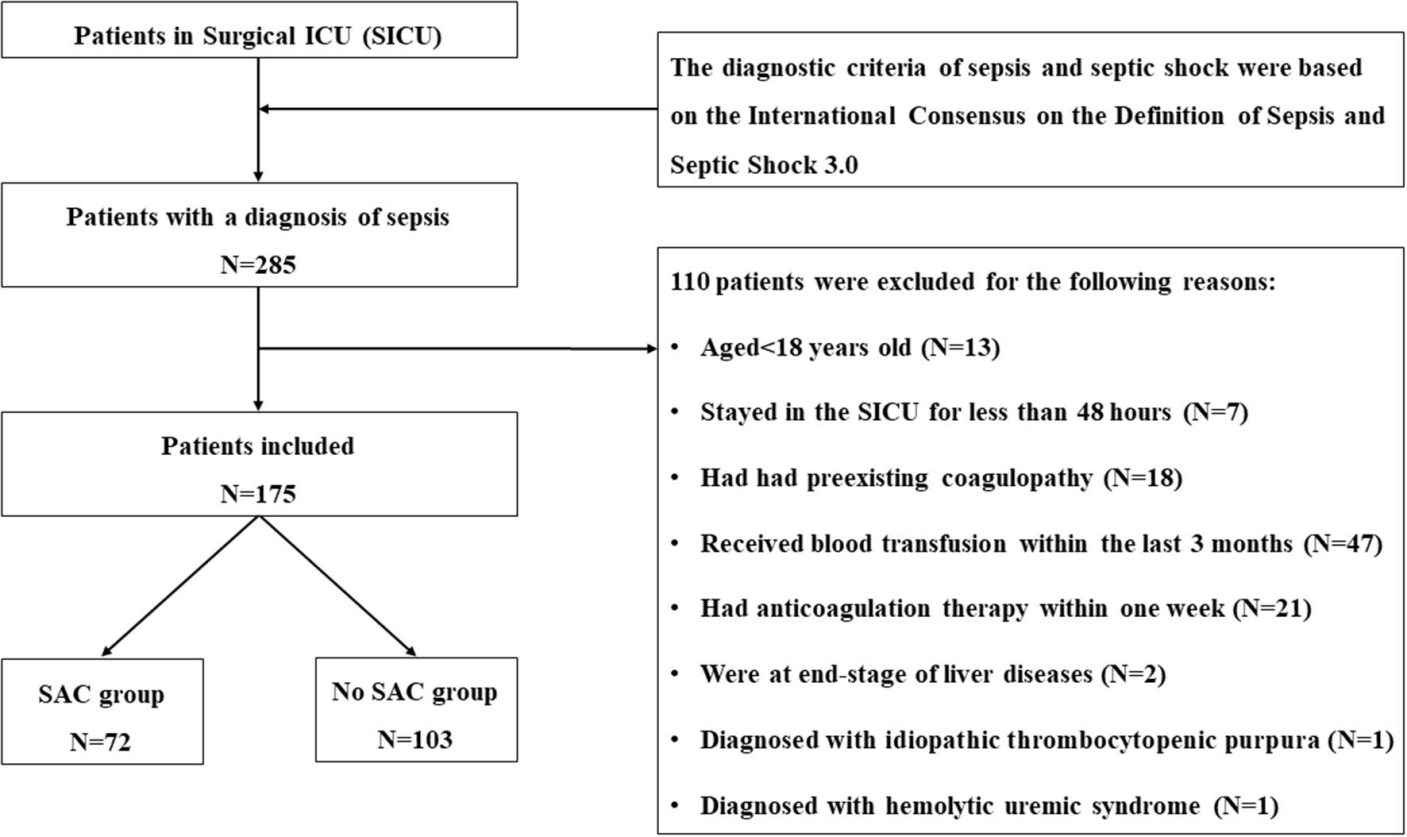
Flow diagram of patient inclusion.

During the study period, 285 patients were admitted to surgical ICU with postoperative sepsis, in which 175 of them met the inclusion criteria and were eventually included in the current study. Detailed selection process was summarized in Figure 1. Overall, the median age of the final cohort was 65 (IQR, 53–78) years, 57.1% (100/175) patients were men, and the mean body mass index (BMI) was 23.7 ± 4 kg/m^2^. Among all the included patients, 41.1% (72/175) of ICU patients were identified as having SAC, whereas 58.9% (103/175) of patients did not develop SAC during the ICU stay.

As shown in Table 1, we summarized the baseline characteristics between SAC and no SAC groups. There were statistically significant differences in age [72 (IQR, 56–81) vs. 61.0 (IQR, 49–74); *p* = 0.012] and comorbidities, including hypertension [30.6% (22/72) vs. 46.6% (48/103); *p* = 0.042] and CHF [8.3% (6/72) vs. 1% (1/103); *p* = 0.02] between two groups. Additionally, patients with SAC had significantly higher levels of creatinine [120.40 (IQR, 86.8–193.0) vs. 91.6 (IQR, 61.3–127.8); *p* < 0.001], BUN [12 (IQR, 8.7–19.1) vs. 8.1 (IQR, 4.7–11.5); *p* < 0.001], AST [49.6 (IQR, 25.6–162.6) vs. 28.5 (IQR, 19.2–56.2); *p* < 0.001], INR [1.7 (IQR, 1.5–2) vs. 1.2 (IQR, 1.1–1.3); *p* < 0.001], IL-6 [98.7 (IQR, 38.2–379.3) vs. 55 (IQR, 24.60–117.4); *p* = 0.002], and potassium [3.9 (IQR, 3.7–4.2) vs. 3.7 (IQR, 3.5–4.1), *p* = 0.009]. Of note, severity of illness indicated by SOFA, APACHE II, and the need of renal replacement therapy, mechanical ventilation, and blood transfusion was also found to correlate with the development of SAC. No significant differences were observed in gender, BMI, the development of comorbidities (diabetes, CHD, cerebral infarction, COPD, CRI, and malignant neoplasm), source of infection, total bilirubin, ALT, hemoglobin, WBC, CRP, procalcitonin, sodium, tracheotomy, deep vein catheterization, and hospital LOS between the two groups.

**Table 1:** Baseline characteristics of included patients stratifying by the presence of sepsis-associated coagulopathy (SAC).

**Characteristics**	**Total (*n =* 175)**	**SAC (*n =* 72)**	**No SAC (*n =* 103)**	***P*-value**
**Demographics**	
Age, years, median (IQR)	65.0 (53.0–78.0)	72.0 (56.0–81.0)	61.0 (49.0–74.0)	**0.012**
Gender, male, *n* (%)	100 (57.1)	42 (58.3)	58 (56.3)	0.790
BMI, kg/m^2^, mean (SD)	23.7 (4.0)	23.6 (4.5)	23.7 (3.7)	0.093
**Comorbidities**, ***n*** **(%)**
Hypertension	60 (34.3)	22 (30.6)	48 (46.6)	**0.042**
Diabetes	46 (26.3)	21 (29.2)	25 (24.3)	0.469
CHD	25 (14.3)	13 (18.1)	12 (11.7)	0.233
Cerebral Infarction	26 (14.9)	10 (13.9)	16 (15.5)	0.763
COPD	9 (5.1)	6 (8.3)	3 (2.9)	0.164
CRI	21 (12.0)	7 (9.7)	14 (13.6)	0.438
CHF	7 (4.0)	6 (8.3)	1 (1.0)	**0.020**
Malignant neoplasm	12 (6.9)	3 (4.2)	9 (8.7)	0.364
**Source of infection**, ***n*** **(%)**				0.380
Pulmonary	57 (32.6)	28 (38.9)	29 (28.2)	
Abdominal	69 (39.4)	27 (37.5)	42 (40.8)	
Genitourinary	31 (17.7)	11 (15.3)	20 (19.4)	
Skin or soft tissue	7 (4.0)	1 (1.4)	6 (5.8)	
Unknown	11 (6.3)	5 (6.9)	6 (5.8)	
**Laboratory findings and blood gas analysis**	
INR, median (IQR)	1.3 (1.2–1.6)	1.7 (1.5–2.0)	1.2 (1.1–1.3)	**<0.001**
Albumin, mg/dL, median (IQR)	29.2 (25.4–32.4)	27.7 (23.8–30.4)	30.9 (26.7–33.8)	**<0.001**
Total bilirubin (umol/L), median (IQR)	16.6 (11.4–36.8)	18.9 (11.5–42.8)	15.3 (11.2–34.4)	0.280
ALT (U/L), median (IQR)	29.2 (14.4–75.5)	35.1 (14.4–117.7)	27.7 (14.2–48.7)	0.148
AST (U/L), median (IQR)	37.6 (20.8–74.2)	49.6 (25.6–162.6)	28.5 (19.2–56.2)	**<0.001**
Creatinine, μmol/L, median (IQR)	99.0 (71.7–158.6)	120.40 (86.8–193.0)	91.6 (61.3–127.8)	**<0.001**
BUN, mg/dL, median (IQR)	9.5 (6.1–15.8)	12.0 (8.7–19.1)	8.1 (4.7–11.5)	**<0.001**
Hemoglobin, g/dL, mean (SD)	103.2 (20.8)	100.1 (20.2)	105.3 (21.0)	0.102
RBC, 10^12^/L, mean (SD)	3.4 (0.7)	3.3 (0.6)	3.5 (0.7)	**0.048**
Platelet, 10^9^/L, median (IQR)	94.0 (49.0–167.0)	80.5 (39.5–107.5)	124.0 (61.0–199.0)	**<0.001**
WBC, 10^9^/L, median (IQR)	11.2 (7.2–17.9)	12.0 (7.1–19.9)	11.0 (7.2–16.8)	0.219
CRP (mg/L), median (IQR)	10.3 (6.8–15.3)	12.0 (7.1–16.4)	9.5 (6.6–14.1)	0.058
Procalcitonin (ng/ml), median (IQR)	11.9 (2.6–62.1)	20.9 (3.9–72.6)	8.8 (2.3–33.1)	0.057
IL-6 (pg/ml), median (IQR)	78.0 (32.3–203.0)	98.7 (38.2–379.3)	55.0 (24.60–117.4)	**0.002**
Potassium, mmol/L, median (IQR)	3.8 (3.5–4.1)	3.9 (3.7–4.2)	3.7 (3.5–4.1)	**0.009**
Sodium, mmol/L, mean (SD)	140.8 (7.5)	142.0 (7.8)	140.0 (7.2)	0.082
**Prognostic scoring systems, median (IQR)**	
SOFA	6.0 (4.0–9.0)	8.0 (6.0–9.0)	5.0 (4.0–7.0)	**<0.001**
APACHE II	13.0 (9.0–16.0)	15.0 (13.0–18.0)	11.0 (8.0–15.0)	**<0.001**
**Clinical interventions (** ***n*** **, %)**	
Mechanical ventilation, *n* (%)	49 (28.0)	27 (37.5)	22 (21.4)	**0.019**
Renal replacement therapy, *n* (%)	94 (53.7)	47 (65.3)	47 (45.6)	**0.010**
Tracheotomy, *n* (%)	9 (5.1)	5 (6.9)	4 (3.9)	0.491
Deep vein catheterization, *n* (%)	143 (81.7)	63 (87.5)	80 (77.7)	0.098
Blood transfusion, *n* (%)	85 (48.6)	52 (72.2)	33 (32.0)	**<0.001**
**Clinical outcomes**	
Hospital mortality, *n* (%)	39 (22.3)	27 (37.5)	12 (11.7)	**<0.001**
Septic shock, *n* (%)	39 (22.3)	31 (43.1)	8 (7.8)	**<0.001**
LOS, median (IQR)	11.0 (6.0–21.0)	12.5 (6.0–23.8)	10.0 (6.0–19.0)	0.397

*Abbreviations: SAC, sepsis associated coagulopathy; IQR, interquartile range; CHD, coronary heart disease; COPD, chronic obstructive pulmonary disorder; CRI, chronic renal insufficiency; CHF, chronic heart failure; INR, international normalized ratio; SOFA, sequential organ failure assessment; APACHE II, Acute Physiology and Chronic Health Evaluation II; LOS, length of stay. The bold values indicate the P values with significant difference*.

### Relationship Between SAC and Clinical Outcomes

**Figure 2:**
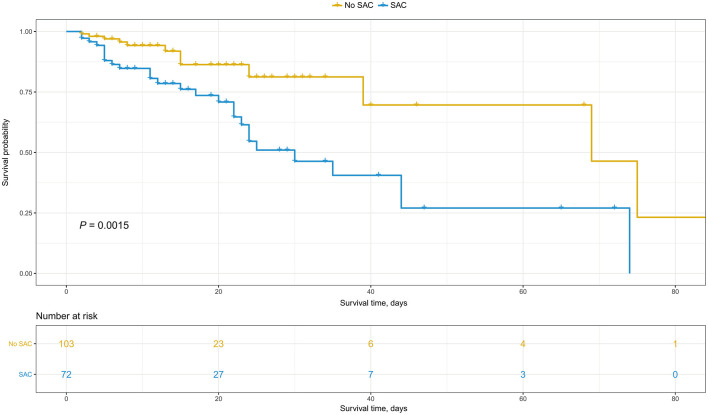
Kaplan-Meier analysis for cumulative survival between sepsis-associated coagulopathy (SAC) and No SAC groups.

Overall, 37.5% (27/72) patients in the SAC group and 11.7% (12/103) patients in the no SAC group died during hospitalization. Likewise, Kaplan-Meier analysis combined with log-rank test indicated the greater in-hospital mortality among patients with SAC compared with those without SAC (*p* = 0.002) (Figure 2). Univariable and multivariable Cox regression models were subsequently constructed to explore the association between SAC and in-hospital mortality. Univariable Cox regression analysis revealed that presence of SAC was significantly associated with an increased in-hospital mortality for patients with sepsis admitted to surgical ICU [Hazard ratio (HR), 3.75; 95% Confidence interval (CI), 1.90–7.40; *p* < 0.001] (Table 2 and Supplementary Table S1). Similarly, by performing multivariable analysis, significant differences were also observed with respect to in-hospital mortality (HR, 2.39; 95% CI, 1.15–6.15; *p* = 0.023) after adjusting for possible confounding factors (Table 2 and Supplementary Table S1).

**Table 2:** Univariable and multivariable analyses of SAC in predicting clinical outcomes.

**Outcomes**	**SAC (*n =* 72)**	**No SAC (*n =* 103)**	**HR/OR (95%CI)**	***P*-value**
**Primary outcome**	
Hospital mortality	27 (37.5)	12 (11.7)		**<0.001**
Univariable analysis			3.75 (1.90–7.40)	**<0.001**
Multivariate analysis			2.39 (1.15–6.15)	**0.023**
**Secondary outcomes**	
Septic shock	31 (43.1)	8 (7.8)		**<0.001**
Univariable analysis			7.41 (3.76–14.61)	**<0.001**
Multivariate analysis			4.11 (1.81–9.32)	**0.001**
LOS	12.5 (6.0–23.8)	10.0 (6.0–19.0)		0.397
Univariable analysis			1.08 (0.93–1.26)	0.306
Multivariate analysis			0.97 (0.83–1.14)	0.743

*HR, Hazard ratio; OR, Odds ratio; LOS, length of stay. The bold values indicate the P values with significant difference*.

As for the secondary endpoints, univariable and multivariable logistic and linear regression models were performed to identify the correlation of SAC with the development of septic shock and hospital LOS, respectively (Table 2 and Supplementary Tables S2, S3). Identical to the primary endpoint, a complication of SAC in surgical ICU patients was found to be an independent predictor for the development of septic shock [Odds Ratio (OR), 4.11; 95% CI, 1.81–9.32; *p* = 0.001]. Nevertheless, the presence of SAC was not significantly associated with a prolonged hospital stay (OR, 0.97; 95% CI, 0.83–1.14; *p* = 0.743).

### Sensitivity and Subgroup Analyses

**Figure 3:**
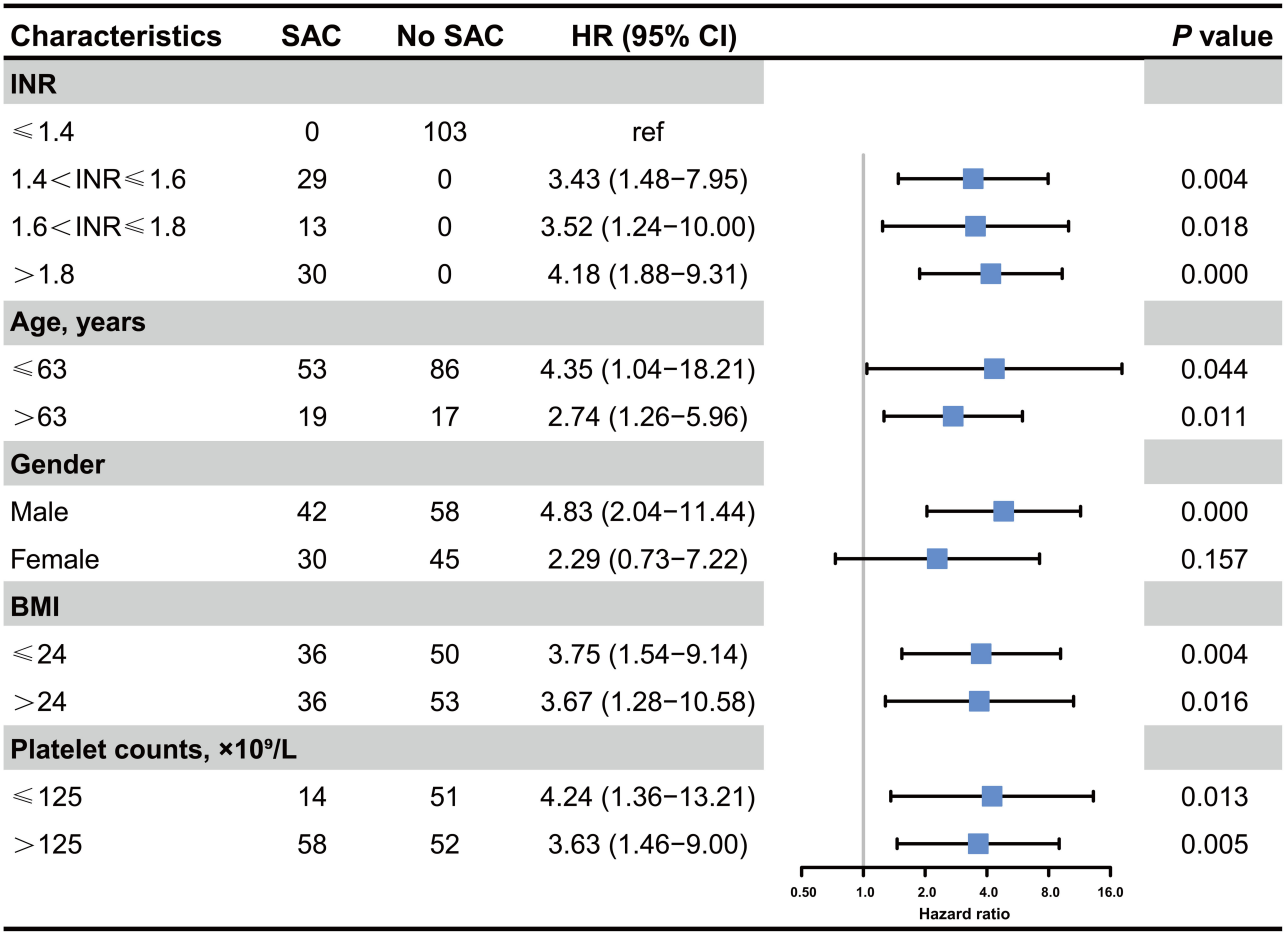
Association between the presence of SAC and in-hospital mortality in critically ill patients with postoperative sepsis. HR, Hazard ratio; INR, international normalized ratio; BMI, Body mass index.

Furthermore, we performed a series of sensitivity analyses to validate the robustness of our findings (Figure 3). By stratifying severity of SAC based on INR value, we consistently demonstrated that mild (HR, 3.43; 95% CI, 1.48–7.95; *p* = 0.004), moderate (HR, 3.52; 95% CI, 1.24–10.00; *p* = 0.018), and severe SAC (HR, 4.18; 95% CI, 1.88–9.31; *p* < 0.001) were significantly correlated with an increased in-hospital mortality of patients with sepsis. Furthermore, patients with more severe SAC had higher risk of in-hospital death. Likewise, the results remained unchanged by stratifying patients with age, BMI, and platelet count. Intriguingly, when taking gender into consideration, we noticed that male patients with SAC had an increased risk of death during hospitalization compared to those without SAC (HR, 4.83; 95% CI, 2.04–11.44, *p* < 0.001), whereas SAC were not associated with an increased in-hospital mortality in female subgroup (HR, 2.29; 95% CI, 0.73–7.22, *p* = 0.157).

## Discussion

In the current study, we found that the presence of SAC was significantly associated with an increased risk of in-hospital death among patients with postoperative sepsis admitted to ICU. Meanwhile, we identified an increased risk of death in patients with more severe SAC (e.g., patients with moderate SAC has a higher risk of death compared to mild SAC), especially for patients with severe SAC. Meanwhile, based on our analysis, postoperative patients complicated with SAC were prone to develop septic shock compared to those without SAC. Nevertheless, no significantly prolonged hospital LOS has been observed in SAC group.

Of note, a large-scale, single-center, retrospective study reported that the development of SAC identified that patients with severe SAC showed a greater risk of in-hospital death, which showed the same tendency with the results of our study (7). Nevertheless, they also revealed a positive correlation between an SAC severity and a prolonged hospital and ICU LOS. The discrepancy might be attributed to the disparate populations and distinct criteria of diagnosing and stratifying SAC. Since the overall patients in our study were relatively mild with lower APACHE II score and mortality rates in comparison with patients who were incorporated in Lyons's study, the hospital LOS was not long enough to detect the statistical differences. Furthermore, we also found that the presence of SAC was significantly associated with increased risk of septic shock. Correspondingly, Jhang et al. (15) also found that the critically ill pediatric patients complicated with SAC exhibited a high incidence of septic shock. Sepsis-induced hyperinflammatory response was responsible for the increased exposure to tissue factors, followed by an excessive mobilization of coagulation cascade and subsequent generation of thrombin (8). Furthermore, anticoagulant pathways were also impaired by overproduction of proinflammatory cytokines, thereby resulting in the development of SAC (16, 17). Meanwhile, surgical insults were demonstrated to augment the imbalance between coagulation and anticoagulant pathways, leading to the upregulation of plasminogen activator inhibitor with subsequent hyperfibrinolysis (18, 19). Moreover, severe SAC could give rise to the formation of microvascular clots and disseminated intravascular coagulation (DIC), which inevitably caused tissue ischemia and hemodynamic changes, including hypovolemia and myocardial depression, in association with the occurrence of septic shock and irreversible organ dysfunction (9, 20, 21).

Notably, our work has several clinical implications for the management of sepsis. Based on our analysis, we implied that the development of SAC could serve as a marker of sepsis severity due to its latent value in predicting the onset of septic shock and in-hospital mortality. Accordingly, our previous study, which used the Medical Information Mart for Intensive Care (MIMIC-III) database, has validated these results externally (22). By applying the machine learning-based algorithm, we identified the coagulopathy as the second most significant feature for predicting the in-hospital death among critically ill patients with postoperative sepsis. Given that, the current study revealed that a group of patients with SAC were at higher risk of in-hospital death, prompting us to recognize and to interfere the coagulopathy among postoperative patients in the early stage. In addition, several studies have demonstrated that early application of rotational thromboelastometry (ROTEM) and thrombelastography (TEG) might benefit these patients (23, 24).

More importantly, identification of SAC might potentially influence the therapeutic strategies for patients with sepsis. Since both platelets and endothelium played pivotal roles in the development of coagulopathy and septic shock, selective modulation might attenuate the vicious cycle and improve the clinical outcomes. Given that, a phase 2a randomized clinical trial (RCT) incorporating 24 patients showed that the coadministration of iloprost and eptifibatide could significantly reduce fibrinolytic biomarkers and platelet consumption, thereby ameliorating an elevated SOFA score in patients with septic shock (25). Meanwhile, a phase 2b multicenter RCT, involving 750 septic patients with DIC, reported that the 28-day mortality rate was not significantly decreased in patients receiving ART-123 (a recombinant human soluble thrombomodulin), compared to that of the placebo group (26). However, a subsequent *post hoc* analysis revealed that the subgroup of patients with coagulopathy (PT-INR >1.4 at baseline with a platelet count of 30–150 × 10^9^/L) benefited the most from ART-123 administration, for which the 28-day mortality was significantly decreased in the ART-123 group (26). Likewise, a meta-analysis of RCTs comparing anticoagulant therapy with placebo/no intervention in patients with sepsis enrolled 24 trials with 14,767 patients and showed no significantly reduced mortality in overall patients with sepsis, whereas a significant reduction in mortality rate has been observed in the population with a sepsis-induced DIC (27). The results suggested that patients with sepsis with SAC might be the most optimal candidate for studying the clinical efficacy of agents that could influence the coagulation cascade in sepsis. Nevertheless, results from s recently published SCARLET randomized clinical trial enrolling 946 participants from 159 sites indicated that the administration of human recombinant thrombomodulin (rhTM) did not significantly reduce the 28-day all-cause mortality among patients with SAC (14, 28). The results of our study suggested that secondary analyses of published RCTs on postoperative patients with SAC might be favorable for testifying the effectiveness of various agents targeting the coagulation cascade.

Several limitations should be taken into account when interpretating our findings. Firstly, our study was in a single center and retrospective design with a relatively small sample size, which restricted us from detecting a causal relationship. Therefore, well-designed multicenter prospective cohorts are needed to further validate our findings. Secondly, we defined SAC in accordance with the criteria which were proposed by Vincent et al., and we categorized the severity of SAC solely based on INR value rather than platelet counts. Meanwhile, we did not identify the patients with sepsis-induced DIC, which was also a well-known standard for determining the coagulation abnormality. The disparate selection of coagulopathy criteria might potentially alter the results, and their relationship with clinical outcomes required further investigation. Thirdly, we exclusively incorporated the postoperative patients who were admitted to the ICU after surgery. Therefore, our findings might not pertain to relatively mild cases without the ICU stay. Finally, there were several confounders that we were unable to fully measure due to an incomprehensive medical history. These variables might affect the correlation between SAC and outcomes. Meanwhile, many other endpoints were not taken into consideration in the current study. For example, a disparate phase of mortality (ICU mortality, 28-day, and 30-day mortality), alteration of SOFA score (Delta SOFA), and various complications were all well-established candidates to assess the effects of SAC on multiple organ dysfunction and prognosis.

## Conclusions

In conclusion, the development of SAC was associated with an in-hospital mortality and septic shock in critically ill patients with postoperative sepsis. Meanwhile, patients with severe SAC appeared to have a higher risk of death in comparison to those with mild to moderate SAC.

## Data Availability Statement

The original contributions presented in the study are included in the article/Supplementary Material, further inquiries can be directed to the corresponding author/s.

## Ethics Statement

The studies involving human participants were reviewed and approved by the Committee on the Ethics of Medicine of Chinese PLA General Hospital. The patients/participants provided their written informed consent to participate in this study.

## Author Contributions

X-hD, R-qY, and CR conceived the analysis. Y-xL, D-mX, and L-yZ extracted all data. P-yZ and S-yZ undertook and refined the inclusion process. R-qY, CR, and Y-xL co-wrote the paper. R-qY, CR, and D-mX undertook the statistical analyses. X-hD, P-yZ, and L-pL were consulted for clinical issues. All authors contributed to and revised the final manuscript.

## Funding

This work was supported by grants from the National Natural Science Foundation of China (Nos. 81730057, 81801935, and 81930057) and the Key Project of Military Medical Innovation Program of Chinese PLA (No. 18CXZ026).

## Conflict of Interest

The authors declare that the research was conducted in the absence of any commercial or financial relationships that could be construed as a potential conflict of interest.

## Publisher's Note

All claims expressed in this article are solely those of the authors and do not necessarily represent those of their affiliated organizations, or those of the publisher, the editors and the reviewers. Any product that may be evaluated in this article, or claim that may be made by its manufacturer, is not guaranteed or endorsed by the publisher.
