# Supplementary material for: Sepsis-Associated Coagulopathy Predicts Hospital Mortality in Critically Ill Patients With Postoperative Sepsis
Source: Front Med (Lausanne). 2022 Feb 15;9:783234. doi: 10.3389/fmed.2022.783234 (PMC8885730; doi:10.3389/fmed.2022.783234)
Supplement: Supplementary file 1 [file Data_Sheet_1.docx]

**Table S1.** Univariable and multivariate Cox regression analysis of factors associated with hospital mortality

| **Characteristics** | **Univariable HR (95% CI)** | | | ***P* value** | **Multivariable HR (95% CI)** | ***P* value** |
| --- | --- | --- | --- | --- | --- | --- |
| **Demographics** |  | | |  |  |  |
| Age, years | 1.05 (1.03-1.08) | | | **<0.001** | 1.03 (1.00-1.06) | 0.072 |
| Gender |  | | |  |  |  |
| female | 1 | | |  |  |  |
| male | 1.76 (0.89-3.48) | | | 0.102 |  |  |
| BMI, kg/m^2^ | 0.91 (0.85-0.99) | | | **0.025** | 0.94 (0.87-1.02) | 0.157 |
| SAC | 3.75 (1.90-7.40) | | | **<0.001** | 2.39 (1.15-6.15) | **0.023** |
| **Comorbidities** |  | | |  |  |  |
| Hypertension | 1.16 (0.62-2.19) | | | 0.638 |  |  |
| Diabetes | 1.26 (0.60-2.66) | | | 0.541 |  |  |
| CHD | 3.12 (1.58-6.17) | | | **0.001** | 2.24 (0.96-5.23) | 0.061 |
| Cerebral Infarction | 1.58 (0.73-3.43) | | | 0.250 |  |  |
| COPD | 1.05 (0.25-4.37) | | | 0.943 |  |  |
| CRI | 1.42 (0.60-3.39) | | | 0.429 |  |  |
| CHF | 2.13 (0.66-6.92) | | | 0.209 |  |  |
| Malignant neoplasm | 1.18 (0.36-3.84) | | | 0.780 |  |  |
| **Source of infection** |  | | |  |  |  |
| Pulmonary | 2.29 (0.54-9.77) | | | 0.263 | 0.93 (0.20-4.34) | 0.928 |
| Abdominal | 0.88 (0.20-3.97) | | | 0.869 | 0.50 (0.11-2.35) | 0.378 |
| Genitourinary | 0.69 (0.13-3.75) | | | 0.665 | 0.37 (0.06-2.20) | 0.274 |
| Skin or soft tissue | 0.83 (0.08-9.15) | | | 0.829 | 1.34 (0.12-15.27) | 0.811 |
| Unknown | reference | | |  |  |  |
| **Laboratory findings and blood gas analysis** | | |  |  |  |  |
| INR | 1.17 (0.93-1.46) | | | 0.177 | 0.98 (0.61-1.57) | 0.924 |
| Albumin, mg/dL | 0.99 (0.94-1.05) | | | 0.801 |  |  |
| Total bilirubin, umol/L | 1.00 (0.99-1.01) | | | 0.648 |  |  |
| ALT, U/L | 1.00 (1.00-1.00) | | | 0.102 |  |  |
| AST, U/L | 1.00 (1.00-1.00) | | | **0.002** | 1.00 (1.00-1.00) | 0.051 |
| Creatinine, μmol/L | 1.00 (1.00-1.00) | | | **0.007** | 1.00 (1.00-1.01) | 0.071 |
| BUN, mg/dL | 1.05 (1.02-1.08) | | | **0.003** | 0.99 (0.94-1.03) | 0.502 |
| Hemoglobin, g/dL | 1.00 (0.98-1.01) | | | 0.607 |  |  |
| RBC, 10^12^/L | 0.98 (0.60-1.58) | | | 0.922 |  |  |
| Platelet, 10^9^/L | 1.00 (1.00-1.00) | | | 0.526 |  |  |
| WBC, 10^9^/L | 1.00 (0.99-1.01) | | | 0.699 |  |  |
| CRP, mg/L | 1.02 (0.97-1.06) | | | 0.474 |  |  |
| Procalcitonin, ng/ml | 0.99 (0.98-1.00) | | | **0.056** | 0.99 (0.98-1.00) | **0.030** |
| IL-6, pg/ml | 1.00 (1.00-1.00) | | | **0.002** | 1.00 (1.00-1.00) | 0.158 |
| Potassium, mmol/L | 0.80 (0.41-1.59) | | | 0.528 | 0.70 (0.34-1.41) | 0.312 |
| Sodium, mmol/L | 1.02 (0.98-1.07) | | | 0.260 |  |  |
| **Prognostic scoring systems, median (IQR)** | |  | |  |  |  |
| SOFA | 1.12 (1.02-1.23) | | | **0.022** | 1.03 (0.88-1.21) | 0.712 |
| APACHE II | 1.15 (1.09-1.22) | | | **<0.001** | 1.06 (0.97-1.16) | 0.225 |

Abbreviation: SAC, sepsis associated coagulopathy; CHD, coronary heart disease; COPD, chronic obstructive pulmonary disorder; CRI, chronic renal insufficiency; CHF, chronic heart failure; INR, international normalized ratio; SOFA, sequential organ failure assessment; APACHE II, Acute Physiology and Chronic Health Evaluation II.

**Table S2.** Univariable and multivariable logistic regression analysis for septic shock

| **Characteristics** | **Univariable OR (95% CI)** | | | ***P* value** | **Multivariable OR (95% CI)** | ***P* value** |
| --- | --- | --- | --- | --- | --- | --- |
| **Demographics** |  | | |  |  |  |
| Age, years | 1.03 (1.01-1.05) | | | **0.001** | 1.04 (1.00-1.07) | **0.030** |
| Gender |  | | |  |  |  |
| female | 1 | | |  |  |  |
| male | 1.20 (0.65-2.20) | | | 0.564 |  |  |
| BMI, kg/m^2^ | 0.94 (0.87-1.02) | | | 0.122 |  |  |
| SAC | 7.41 (3.76-14.61) | | | **<0.001** | 4.11 (1.81-9.32) | **0.001** |
| **Comorbidities** |  | | |  |  |  |
| Hypertension | 0.69 (0.37-1.28) | | | 0.234 |  |  |
| Diabetes | 1.29 (0.65-2.53) | | | 0.470 |  |  |
| CHD | 2.02 (0.86-4.75) | | | 0.107 |  |  |
| Cerebral Infarction | 1.53 (0.66-3.52) | | | 0.322 |  |  |
| COPD | 1.85 (0.48-7.13) | | | 0.373 |  |  |
| CRI | 0.41 (0.14-1.16) | | | **0.093** | 0.52 (0.14-2.00) | 0.344 |
| CHF | 1.96 (0.43-9.04) | | | 0.388 |  |  |
| Malignant neoplasm | 0.45 (0.12-1.74) | | | 0.249 |  |  |
| **Source of infection** |  | | |  |  |  |
| Pulmonary | 0.59 (0.16-2.25) | | | 0.441 |  |  |
| Abdominal | 0.33 (0.09-1.22) | | | 0.096 |  |  |
| Genitourinary | 0.27 (0.06-1.15) | | | 0.077 |  |  |
| Skin or soft tissue | 0.10 (0.01-1.10) | | | 0.060 |  |  |
| Unknown | ref | | |  |  |  |
| **Laboratory findings and blood gas analysis** | | |  |  |  |  |
| Albumin, mg/dL | 0.90 (0.85-0.96) | | | **0.001** | 0.97 (0.90-1.04) | 0.318 |
| Total bilirubin, umol/L | 1.00 (0.99-1.01) | | | 0.901 |  |  |
| ALT, U/L | 1.00 (1.00-1.00) | | | 0.215 |  |  |
| AST, U/L | 1.00 (1.00-1.00) | | | **0.098** | 1.00 (1.00-1.00) | 0.570 |
| Creatinine, μmol/L | 1.00 (1.00-1.00) | | | 0.208 |  |  |
| BUN, mg/dL | 1.06 (1.02-1.10) | | | **0.003** | 1.00 (0.94-1.05) | 0.879 |
| Hemoglobin, g/dL | 0.99 (0.97-1.00) | | | **0.068** | 0.96 (0.92-1.01) | 0.130 |
| RBC, 10^12^/L | 0.63 (0.40-1.02) | | | **0.061** | 2.21 (0.48-10.30) | 0.311 |
| Platelet, 10^9^/L | 1.00 (0.99-1.00) | | | **0.045** | 1.00 (1.00-1.00) | 0.977 |
| WBC, 10^9^/L | 1.00 (0.99-1.01) | | | 0.558 |  |  |
| CRP, mg/L | 1.03 (0.99-1.07) | | | 0.191 |  |  |
| Procalcitonin, ng/ml | 1.00 (0.99-1.01) | | | 0.986 |  |  |
| IL-6, pg/ml | 1.00 (1.00-1.01) | | | **0.006** | 1.00 (1.00-1.00) | 0.124 |
| Potassium, mmol/L | 1.28 (0.68-2.40) | | | 0.450 |  |  |
| Sodium, mmol/L | 1.04 (1.00-1.09) | | | **0.053** | 1.02 (0.97-1.08) | 0.360 |
| **Prognostic scoring systems, median (IQR)** | |  | |  |  |  |
| SOFA | 1.23 (1.10-1.38) | | | **<0.001** | 1.07 (0.88-1.30) | 0.520 |

Abbreviation: CHD, coronary heart disease; COPD, chronic obstructive pulmonary disorder; CRI, chronic renal insufficiency; CHF, chronic heart failure; SOFA, sequential organ failure assessment; APACHE II, Acute Physiology and Chronic Health Evaluation II.

**Table S3.** Univariable and multivariable linear regression analysis for hospital LOS

| **Characteristics** | **Univariable OR (95% CI)** | | | ***P* value** | **Multivariable OR (95% CI)** | ***P* value** |
| --- | --- | --- | --- | --- | --- | --- |
| **Demographics** |  | | |  |  |  |
| Age, years | 1.28 (1.11-1.48) | | | **0.001** | 1.13 (0.96-1.34) | 0.129 |
| Gender |  | | |  |  |  |
| female | reference | | |  |  |  |
| male | 0.93 (0.80-1.08) | | | 0.320 |  |  |
| BMI, kg/m^2^ | 0.94 (0.81-1.09) | | | 0.378 |  |  |
| SAC | 1.08 (0.93-1.26) | | | 0.306 | 0.97 (0.83-1.14) | 0.743 |
| **Comorbidities** |  | | |  |  |  |
| Hypertension | 1.03 (0.88-1.19) | | | 0.744 |  |  |
| Diabetes | 1.21 (1.04-1.40) | | | **0.012** | 1.15 (1.00-1.32) | 0.054 |
| CHD | 1.15 (0.99-1.33) | | | **0.073** | 0.98 (0.85-1.13) | 0.786 |
| Cerebral Infarction | 1.20 (1.03-1.39) | | | **0.017** | 1.07 (0.93-1.24) | 0.332 |
| COPD | 1.09 (0.94-1.26) | | | 0.265 |  |  |
| CRI | 0.95 (0.82-1.11) | | | 0.521 |  |  |
| CHF | 1.09 (0.94-1.27) | | | 0.238 |  |  |
| Malignant neoplasm | 1.03 (0.89-1.20) | | | 0.680 |  |  |
| **Source of infection** |  | | |  |  |  |
| Pulmonary | 1.22 (0.91-1.65) | | | 0.189 |  |  |
| Abdominal | 0.95 (0.69-1.29) | | | 0.720 |  |  |
| Genitourinary | 0.98 (0.75-1.27) | | | 0.853 |  |  |
| Skin or soft tissue | 0.99 (0.83-1.20) | | | 0.952 |  |  |
| Unknown | reference | | |  |  |  |
| **Laboratory findings and blood gas analysis** | | |  |  |  |  |
| Albumin, mg/dL | 1.02 (0.88-1.19) | | | 0.792 |  |  |
| Total bilirubin, umol/L | 0.95 (0.81-1.10) | | | 0.461 |  |  |
| ALT, U/L | 0.87 (0.75-1.01) | | | **0.070** | 0.98 (0.79-1.22) | 0.879 |
| AST, U/L | 0.86 (0.74-1.00) | | | **0.051** | 0.90 (0.73-1.12) | 0.358 |
| Creatinine, μmol/L | 1.02 (0.88-1.19) | | | 0.793 |  |  |
| BUN, mg/dL | 1.09 (0.93-1.26) | | | 0.279 |  |  |
| Hemoglobin, g/dL | 0.90 (0.77-1.04) | | | 0.151 |  |  |
| RBC, 10^12^/L | 0.94 (0.81-1.09) | | | 0.401 |  |  |
| Platelet, 10^9^/L | 1.16 (1.00-1.34) | | | **0.054** | 1.13 (0.98-1.31) | 0.085 |
| WBC, 10^9^/L | 0.95 (0.82-1.11) | | | 0.532 |  |  |
| CRP, mg/L | 0.97 (0.83-1.13) | | | 0.680 |  |  |
| Procalcitonin, ng/ml | 0.95 (0.82-1.11) | | | 0.527 |  |  |
| IL-6, pg/ml | 0.93 (0.80-1.08) | | | 0.357 |  |  |
| Potassium, mmol/L | 0.99 (0.85-1.15) | | | 0.906 |  |  |
| Sodium, mmol/L | 1.01 (0.87-1.17) | | | 0.893 |  |  |
| **Prognostic scoring systems, median (IQR)** | |  | |  |  |  |
| SOFA | 0.96 (0.82-1.11) | | | 0.553 |  |  |
| APACHE II | 1.23 (1.06-1.42) | | | **0.007** | 1.06 (0.89-1.25) | 0.517 |
| **Clinical intervention** |  | | |  |  |  |
| Mechanical ventilation | 1.13 (0.97-1.31) | | | 0.110 |  |  |
| Renal replacement therapy | 1.12 (0.96-1.30) | | | 0.139 |  |  |
| Tracheotomy | 1.34 (1.16-1.55) | | | **<0.001** | 1.25 (1.09-1.44) | **0.002** |
| Deep vein catheterization | 1.09 (0.94-1.27) | | | 0.242 |  |  |
| RBC transfusion | 1.34 (1.16-1.55) | | | **<0.001** | 1.27 (1.09-1.48) | **0.003** |

Abbreviation: SAC, sepsis associated coagulopathy; CHD, coronary heart disease; COPD, chronic obstructive pulmonary disorder; CRI, chronic renal insufficiency; CHF, chronic heart failure; SOFA, sequential organ failure assessment; APACHE II, Acute Physiology and Chronic Health Evaluation II.
